# Metabolomics in Early-Phase Clinical Trials from 2010 to 2025

**DOI:** 10.3390/metabo16100777

**Published:** 2026-10-14

**Authors:** Jack T. Thompson, Maia Megase, Marta Boffito, Mark J. Caulfield, Emma F. Magavern

**Affiliations:** 1 William Harvey Research Institute, Queen Mary University of London, London EC1M 6BQ, UK; m.megase@qmul.ac.uk (M.M.); m.j.caulfield@qmul.ac.uk (M.J.C.); 2 Department of HIV Medicine, Chelsea and Westminster Hospital, London SW10 9NH, UK; marta.boffito@nhs.net; 3 Department of Infectious Disease, Imperial College London, London SW7 2AZ, UK

**Keywords:** metabolomics, metabonomics, metabolite profiling, precision medicine, clinical trial design

## Abstract

**Highlights:**

**What are the main findings?**

- Metabolomics was incorporated into only 640 early-phase clinical trials registered globally between 2010 and 2025, representing approximately 0.35% of all early-phase trials.
- Adoption increased modestly over time but varied substantially by trial phase, geographical location, and therapeutic area.

**What are the implications of the main findings?**

- Despite its potential to support biomarker discovery, mechanistic evaluation, and early assessment of treatment response, metabolomics remains underused in early clinical development.
- Overcoming technical, methodological, and regulatory barriers will be critical to fully realise the role of metabolomics in biomarker-driven, mechanism-informed clinical trial design and precision medicine.

**Abstract:**

**Background/Objectives**: Metabolomics has the potential to enhance the definition of medication response phenotypes in early-phase clinical trials, yet global uptake and distribution are not well characterised. This study provides an international assessment of metabolomics integration in registered early-phase clinical trials. **Methods**: Data were obtained from the World Health Organization’s International Clinical Trials Registry Platform (ICTRP). Early-phase trials (phase 0–II) conducted from 2010 to 2025 were analysed, and metabolomics studies were identified using predefined keyword searches across trial records. **Results**: A total of 183,041 early-phase trials were identified, of which 640 (0.35%) incorporated metabolomics. Uptake has increased modestly over time but remains low overall and is concentrated predominantly in high-income countries. The United Kingdom demonstrated the highest proportional integration (1.3%), while the People’s Republic of China (*n* = 237) and the United States of America (*n* = 90) contributed the greatest absolute number of trials with metabolomics. Among the included metabolomic studies: 187 (29.2%) were phase 0, 229 (35.8%) were phase I trials, 183 (28.6%) were phase II, and 41 (6.4%) were combined phase I/II studies. Metabolomics was most commonly included as a secondary, rather than primary outcome (49.7% vs. 25.3%). **Conclusions**: Metabolomics remains underutilised in early-phase clinical trials. Addressing barriers to implementation will be critical to enable broader integration of metabolomics into early-phase clinical trials and to integrate a multi-omic approach to precision medicine.

## 1. Introduction

Metabolomics, also known as metabonomics or metabolic profiling, is the comprehensive analysis of low-molecular-weight molecules (typically <1 kDa) within a biological sample [1]. It reflects the downstream combined effects of genomic, transcriptomic, and proteomic processes alongside environmental influences such as diet, lifestyle, and gut microbiota, making it a sensitive marker of phenotype [2].

Metabolomics has expanded rapidly, driven by advances in high-throughput analytical platforms capable of detecting an increasing number of low-abundance metabolites [3,4,5,6,7,8]. In parallel, improved bioinformatic methods and public databases have strengthened metabolite identification, while initiatives such as the Metabolomics Standards Initiative have enhanced analytical transparency and reproducibility [9,10,11,12]. Reflecting these advances, the number of metabolomics-related publications indexed in PubMed increased nearly tenfold, from 259 in 2010 to 2522 in 2025 [8].

‘Early-phase’ clinical trials, defined here as phase 0, I, and II interventional studies, represent a particularly promising opportunity for metabolomics [8]. Clinical trial costs are rising, and approximately half of all drugs fail at the phase III stage of clinical development, highlighting the need for enhanced discrimination in early-phase study designs [13,14,15]. Early-phase trials are typically short in duration and involve small numbers of participants, limiting the utility of traditional clinical endpoints. However, these studies offer unparalleled medication response phenotypic depth, often including serial sampling in healthy volunteers under controlled conditions, minimising biological variability, and enabling clearer metabolomic interpretation. In this context, metabolomics enables an exploratory, systems-level assessment of both on-target and off-target drug effects, enabling a more comprehensive understanding of mechanisms underlying efficacy and toxicity [16].

Despite such potential, evidence describing the integration of metabolomics into early-phase clinical trials remains limited. Burt and Nandal (2016) identified 469 metabolomics-related studies registered on ClinicalTrials.gov between 1997 and 2015, of which 166 were interventional drug trials and only 91 were early-phase studies (phase 0–II) [17]. The proportion of registered clinical trials incorporating metabolomics increased from 0.16% in 2004 to a peak of 0.39% in 2014. However, this analysis has not been updated since 2015 and, because it examined a single registry, could not provide a comprehensive assessment of global uptake.

The World Health Organization established the International Clinical Trials Registry Platform (ICTRP) in 2005 to provide a single global access point for clinical trial information [18]. The platform currently aggregates data from 20 national and regional registries that meet predefined standards for data quality, accessibility, governance and technical capacity. Participating registries submit a standardised 24-item Trial Registration Dataset containing information on trial identifiers, titles, recruitment countries, interventions, study design, enrolment dates and outcomes. By combining standardised data from multiple registries, the ICTRP provides a valuable resource for evaluating international uptake of metabolomics.

Using ICTRP data, this study aims to characterise global trends in the adoption of metabolomics in early-phase clinical trials between 2010 and 2025. We examine temporal trends, geographical and registry-level variation, fields of study and whether metabolomics was included as a primary or secondary outcome.

## 2. Materials and Methods

### 2.1. International Clinical Trials Registry Platform Data Extraction

The International Clinical Trials Registry Platform (ICTRP) were contacted for source data and temporary access to the secure ICTRP SharePoint repository was granted on 19 March 2025 following acceptance of the ICTRP data access terms. The full dataset (‘ICTRPFullExport.csv’), uploaded on 5 January 2026, includes all study records up to 31 December 2025 [19].

Data wrangling and analysis were performed in R (version 4.5.1 [13 June 2025]) using RStudio (version 2026.01.0 + 392) [20]. Analyses used the tidyverse (version 2.0.0) suite of packages, along with janitor (version 2.2.1), lubridate (version 1.9.5), and ggrepel (version 0.9.8) [21,22,23,24]. The full code is available in a publicly accessible GitHub repository: https://github.com/Jack-Tandy-Thompson/Metabolomics-in-Early-Phase-Clinical-trials-from-2010-to-2025 (Version 1.0.0). Generative artificial intelligence (ChatGPT-5.5 Plus (OpenAI)) was used to assist with code development, but it did not execute any analysis nor was any record-level data entered into ChatGPT. All code and outputs were reviewed, validated, and refined by the authors [25]. This study conforms with ICTRP data-use requirements.

### 2.2. Study Selection

The analysis was restricted to early-phase interventional clinical studies. Trials were included if the study type contained the term “interventional” and the reported phase corresponded to phase 0, 1, or 2. Studies were further limited to those with enrolment between 2010 and 2025. The study period began in 2010 because most of the registries contributing substantial numbers of records to the ICTRP were operational by this time.

Duplicate records from multiple registry entries were identified based on matching public and scientific titles, excluding records with missing title data. Where such duplicates were identified, a single representative record was retained based on the earliest registration date, consistent with ICTRP guidance on linking related records [26].

### 2.3. Identification of Metabolomic Studies

Metabolomics-related studies were identified through a keyword-based search applied to several data fields. The following terms were used: “metabolom*”, “metabonom*”, “metabolite profil*”, “metabolite fingerprint*”, “small molecule profiling”, “pharmacometabolom*”, “lipidom*”, “glycom*”, “steroidom*”, and “metaboli* phenotyping”, where the asterisk (*) denotes a wildcard allowing for variable word endings. These terms were searched within the scientific title, primary outcomes, secondary outcomes, conditions, and interventions fields. Studies were classified as metabolomic if any of these terms were detected in at least one of the specified fields. The presence of these metabolomics-related terms in the primary and secondary outcome fields was used to determine whether metabolomics was assessed as a primary outcome, a secondary outcome, or both.

### 2.4. Country-Level Analysis

Country-level analyses required standardisation of heterogeneous and inconsistently formatted host country entries. A country lookup table was developed to harmonise naming conventions (provided in the Supplementary Table S10) and countries were grouped according to the United Nations member and observer states [27]. Entries with unidentifiable, non-specific (e.g., continent-level), or incomplete information were categorised as “Missing data” and excluded from the analysis.

For the country-level analysis, both absolute and weighted counts were calculated to account for multinational trials. Absolute counts assigned each trial a count of one in every participating country. Consequently, as many studies were hosted in multiple countries, the overall country count exceeds the number of unique trials. Absolute counts were used to describe the geographical distribution of metabolomic trials and generate a country-level heatmap using Ultimaps [28]. Countries were also grouped into seven continents to summarise absolute counts by region.

Weighted counts divided each trial equally among its participating countries. For example, a trial conducted in four countries contributed 0.25 to each country. This ensured that each trial contributed a total of one across countries, preventing overrepresentation of multinational trials. Weighted counts were used to calculate the proportion of trials involving metabolomics in each country.

### 2.5. Registry-Level Analysis

Registries were identified by their respective standardised trial ID (e.g., trial ID’s starting with ‘NCT’ correspond to the Clinical Trials.gov registry). The European Union Clinical Trials Register (EUCTR) and the Clinical Trials Information System (CTIS) were combined to reflect different generations of the same European Clinical Trial system, resulting in a total of 19 distinct registries included in the analysis.

### 2.6. Field of Study Analysis

Metabolomic studies were manually classified into one of the 15 British National Formulary therapeutic categories according to the field of study that most closely reflected the primary therapeutic purpose of the intervention under investigation.

### 2.7. Statistical Analysis and Visualisation

Descriptive analyses were conducted to assess temporal trends and distributions of metabolomic studies by country, registry, year, phase, outcome, and field of study. Visualisations were generated using the ggplot2 package in R [21]. The datasets resulting from these analyses are provided in full in the Supplementary Information (Supplementary Tables S5–S9).

### 2.8. Manual Audit of Record-Level Classifications

To evaluate the robustness of the search strategy and study classifications, 120 of the 640 included records (18.8%) were manually reviewed. Each record was assessed to determine whether it described metabolomics or metabolite profiling and whether it represented a duplicate registration of the same study. Records were also classified according to whether metabolite profiling was restricted to the investigational drug and its metabolites, as in mass-balance or absorption, distribution, metabolism and excretion (ADME) studies. Where reported, the analytical platform, metabolomics approach (targeted or untargeted), and biological specimen analysed were recorded.

## 3. Results

### 3.1. Identification of Metabolomics-Related Clinical Trials

**Figure 1:**
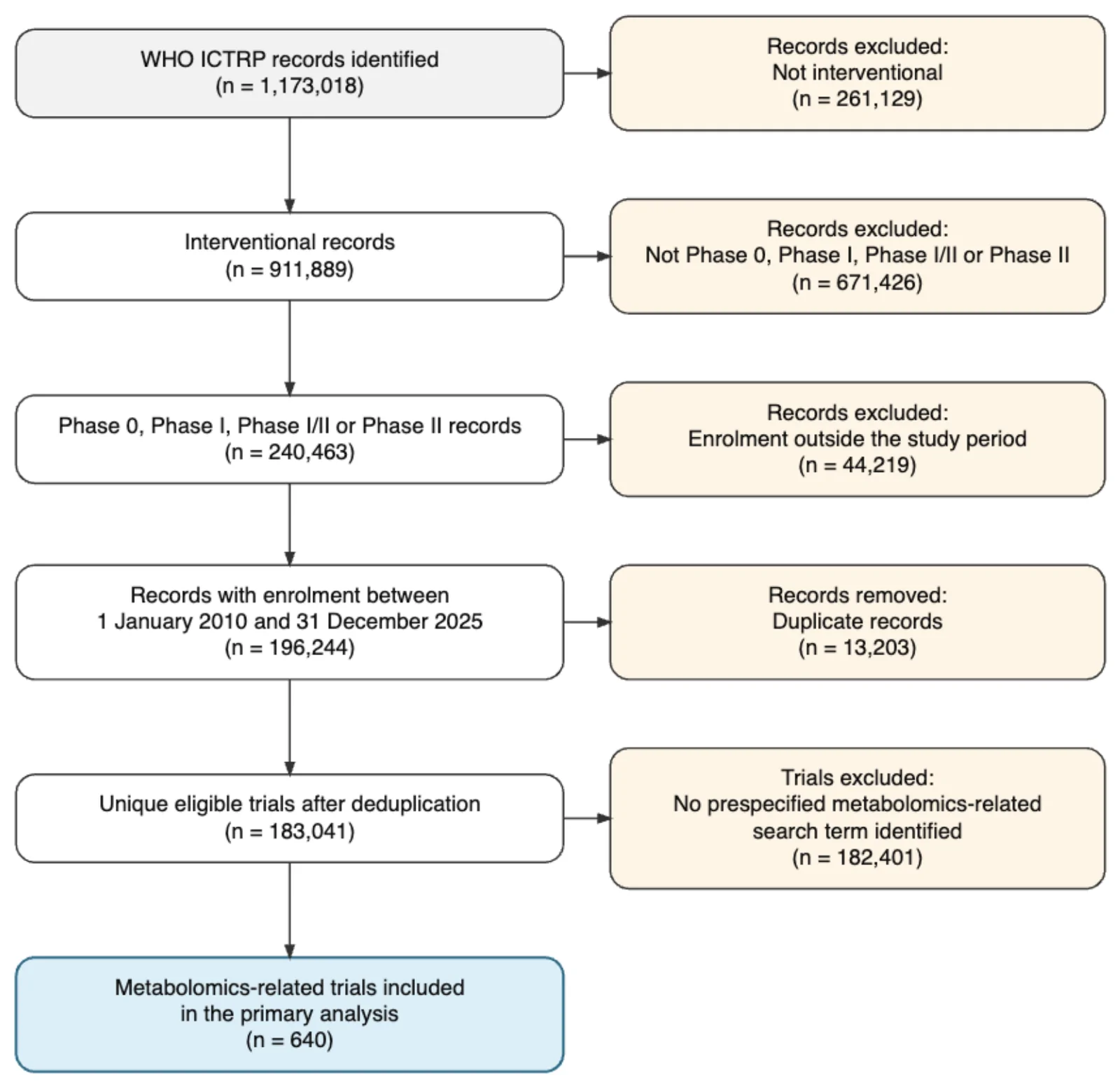
Identification and selection of metabolomics-related early-phase clinical trials from the WHO International Clinical Trials Registry Platform. Early-phase trials were defined as interventional trials classified as Phase 0, Phase I, Phase I/II, or Phase II. Trials were included in the primary analysis when a prespecified metabolomics-related search term was identified in the selected registry fields.

The ICTRP full dataset includes a total of 1,173,018 study records across 63 data fields. In total, 183,041 early-phase clinical trials with enrolment dates between 2010 and 2025 were identified. Of these, 640 studies (0.35%) contained metabolomics-related key terms (Figure 1). The most frequently identified terms were metabolom* (*n* = 419, 65.5%), metabolite profil* (*n* = 152, 23.8%), and metabonom* (*n* = 56, 8.8%). Metabolomics-related terms were most frequently identified under secondary outcomes (*n* = 354, 55.3%), followed by primary outcomes (*n* = 198, 31.0%). A detailed breakdown is provided in the Supplementary Materials (Supplementary Tables S3 and S4).

### 3.2. Geographical Distribution of Metabolomic Studies

**Figure 2:**
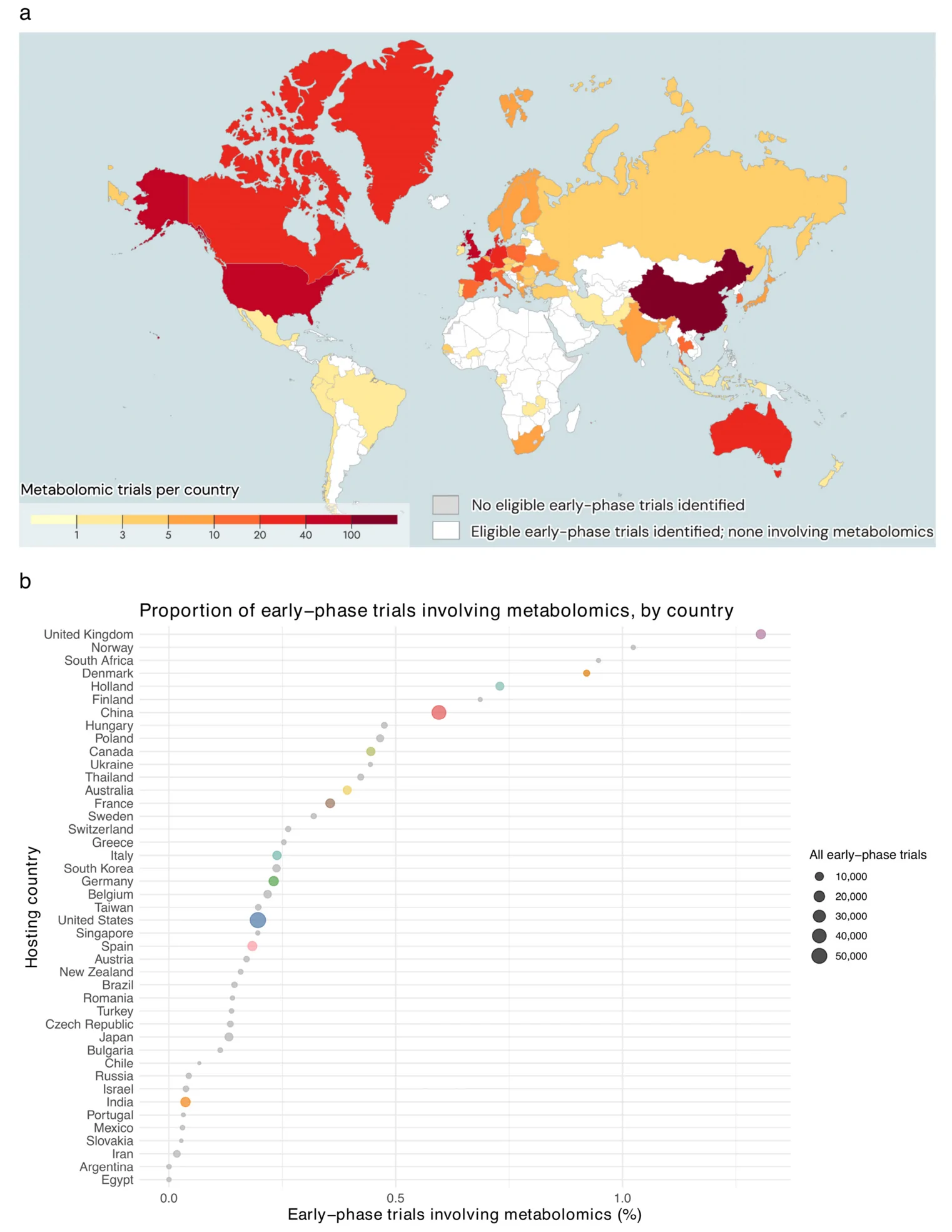
Geographical distribution and country-level proportion of early-phase trials involving metabolomics. (**a**) Global heatmap showing the absolute number of metabolomic trials by country. Each trial is counted once in every participating country. Grey indicates countries with no eligible early-phase clinical trials identified in the ICTRP dataset; white indicates countries with eligible early-phase trials but none involving metabolomics. (**b**) Percentage of eligible early-phase trials involving metabolomics in each country, calculated as the weighted count of metabolomic trials divided by the weighted count of all eligible early-phase trials, multiplied by 100. Weighted counts apportion each trial equally among its participating countries. Bubble size represents the absolute number of eligible early-phase trials in each country. Only countries hosting more than 1000 eligible early-phase trials, based on absolute counts, are included in panel The twelve countries with the largest absolute counts of eligible early-phase trials are highlighted using distinct colours; all remaining countries are shown in grey.

The majority of early-phase trials involving metabolomics are concentrated in a limited number of high-income countries. China hosted the largest number of studies (*n* = 237, 29.1%), followed by the United States (*n* = 90, 11.1%) and the United Kingdom (*n* = 88, 10.8%). Other leading contributors included the Netherlands (*n* = 44, 5.4%), Germany (*n* = 26, 3.2%), France (*n* = 23, 2.8%), Australia (*n* = 21, 2.6%), and Denmark (*n* = 20, 2.5%) (Figure 2a).

On a continental level, most metabolomic studies were conducted in Europe (*n* = 338, 42.8%), Asia (*n* = 296, 37.5%), and North America (*n* = 112, 14.1%), while relatively few were hosted in Oceania (*n* = 23, 2.9%), Africa (*n* = 13, 1.6%), and South America (*n* = 7, 0.9%).

There was substantial variation in the proportion of clinical trials incorporating metabolomics across countries (Figure 2b). Among countries hosting at least 1000 trials, the highest relative uptake normalised to the number of clinical trials nationally was observed in the United Kingdom (1.30%), followed by Norway (1.02%), South Africa (0.95%), Denmark (0.92%), and The Netherlands (0.73%) (Supplementary Table S5).

Among the two countries hosting the largest number of early-phase clinical trials; the United States (53,965 trials) and China (41,675 trials), the United States demonstrated a lower proportional uptake of metabolomics (0.20%) compared with China (0.60%) (Supplementary Table S5).

### 3.3. Longitudinal Trends from 2010 to 2025

**Figure 3:**
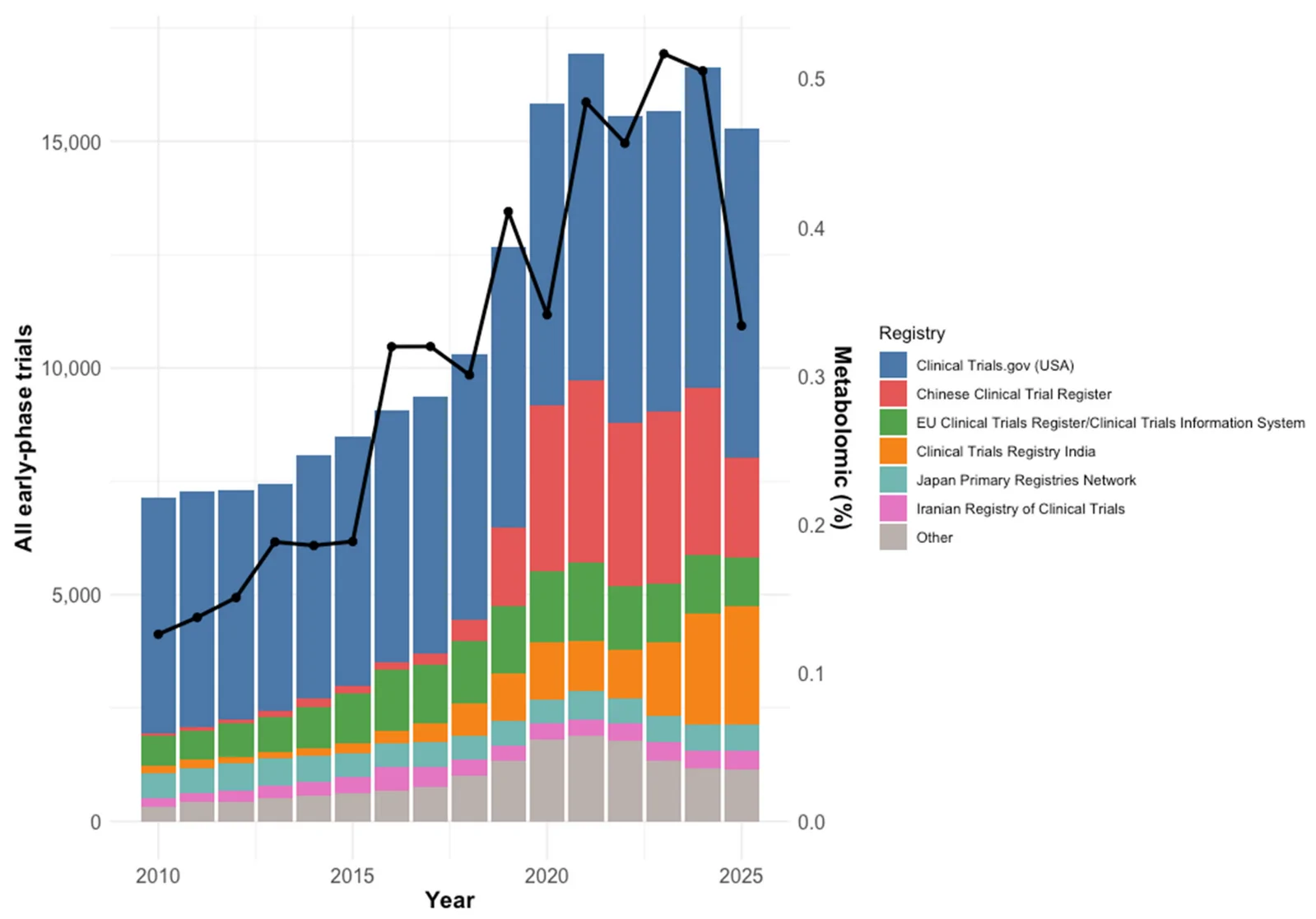
Longitudinal trends in early-phase clinical trials from 2010 to 2025, stratified by registry. Stacked bars show the total number of early-phase trials per year by registry, while the overlaid black line represents the percentage of trials incorporating metabolomics.

The number of early-phase clinical trials, including those incorporating metabolomics, increased over the 16-year study period, with a plateau in overall trial numbers observed from 2021 onwards (Figure 3). Annual early-phase trial numbers rose from 7145 in 2010 to a peak of 16,619 in 2024. Over the same period, the number of trials involving metabolomics increased from 9 in 2010 to 84 in 2024. This corresponded to a rise in the proportion of trials incorporating metabolomics from 0.13% in 2010 to 0.51% in 2024, with a peak of 0.52% observed in 2023.

Almost two thirds (65.7%) of all metabolomic studies found in the ICTRP database were registered with ClinicalTrials.Gov (USA) and the Chinese Clinical Trial Register with much smaller contributions from the other clinical trial registries (Supplementary Table S7).

### 3.4. Metabolomics by Phase

**Figure 4:**
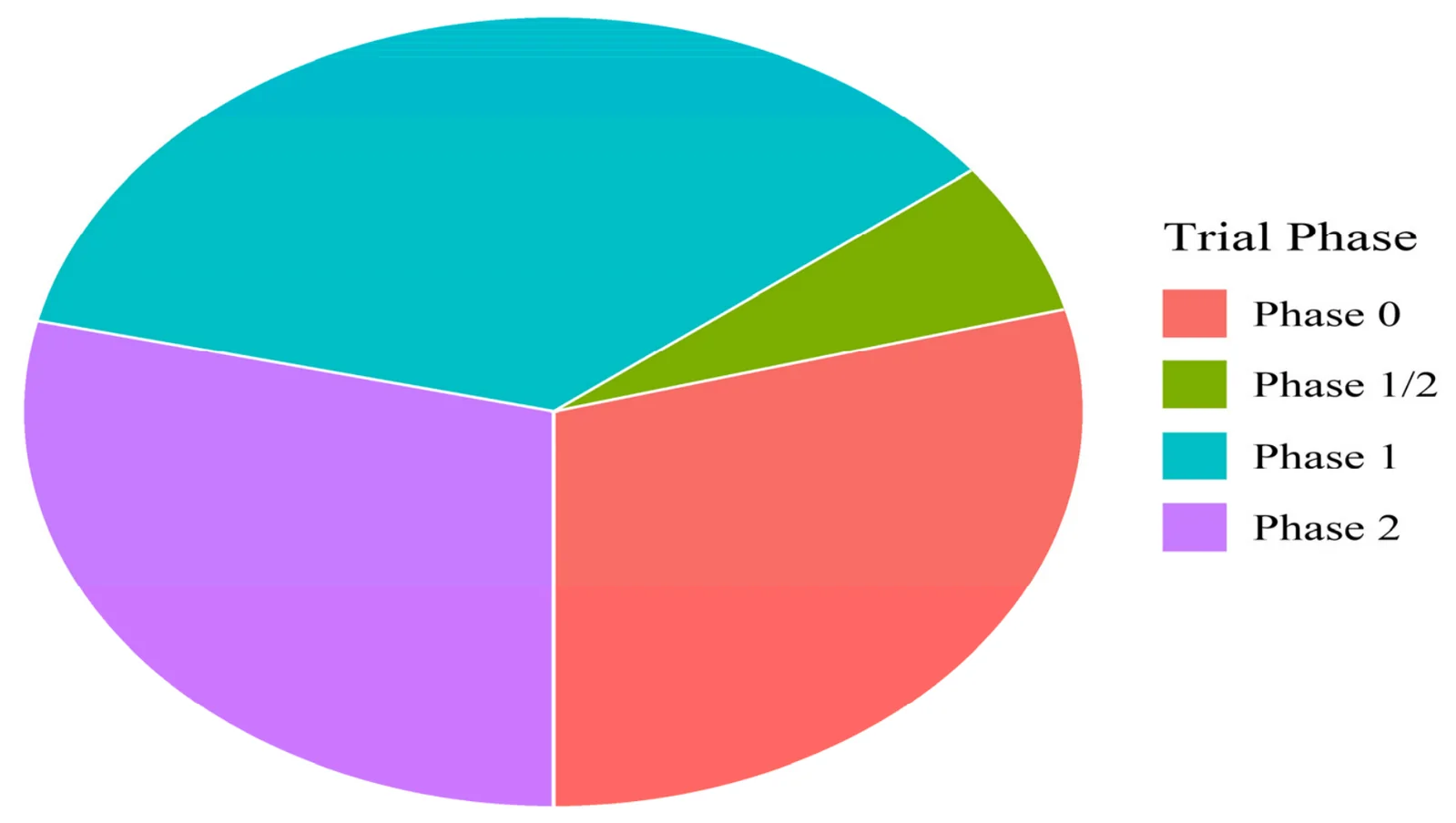
Distribution of metabolomic studies across early clinical trial phases (% of all metabolomic studies).

The distribution of metabolomic studies across early clinical trial phases is shown in Figure 4. Of the included studies, 187 (29.2%) were phase 0, 229 (35.8%) were phase I trials, 183 (28.6%) were phase II, and 41 (6.4%) were combined phase I/II studies.

### 3.5. Metabolomics by Outcome

Metabolomic measures were included as primary outcomes in 25.3% of identified studies (*n* = 162), secondary outcomes in 49.7% (*n* = 318) and both primary and secondary outcomes in 5.6% (*n* = 36). The remaining 19.4% (*n* = 124) did not include metabolomics as a reported outcome measure; however, the ICTRP data do not capture exploratory outcomes.

### 3.6. Metabolomics by Field of Study

**Figure 5:**
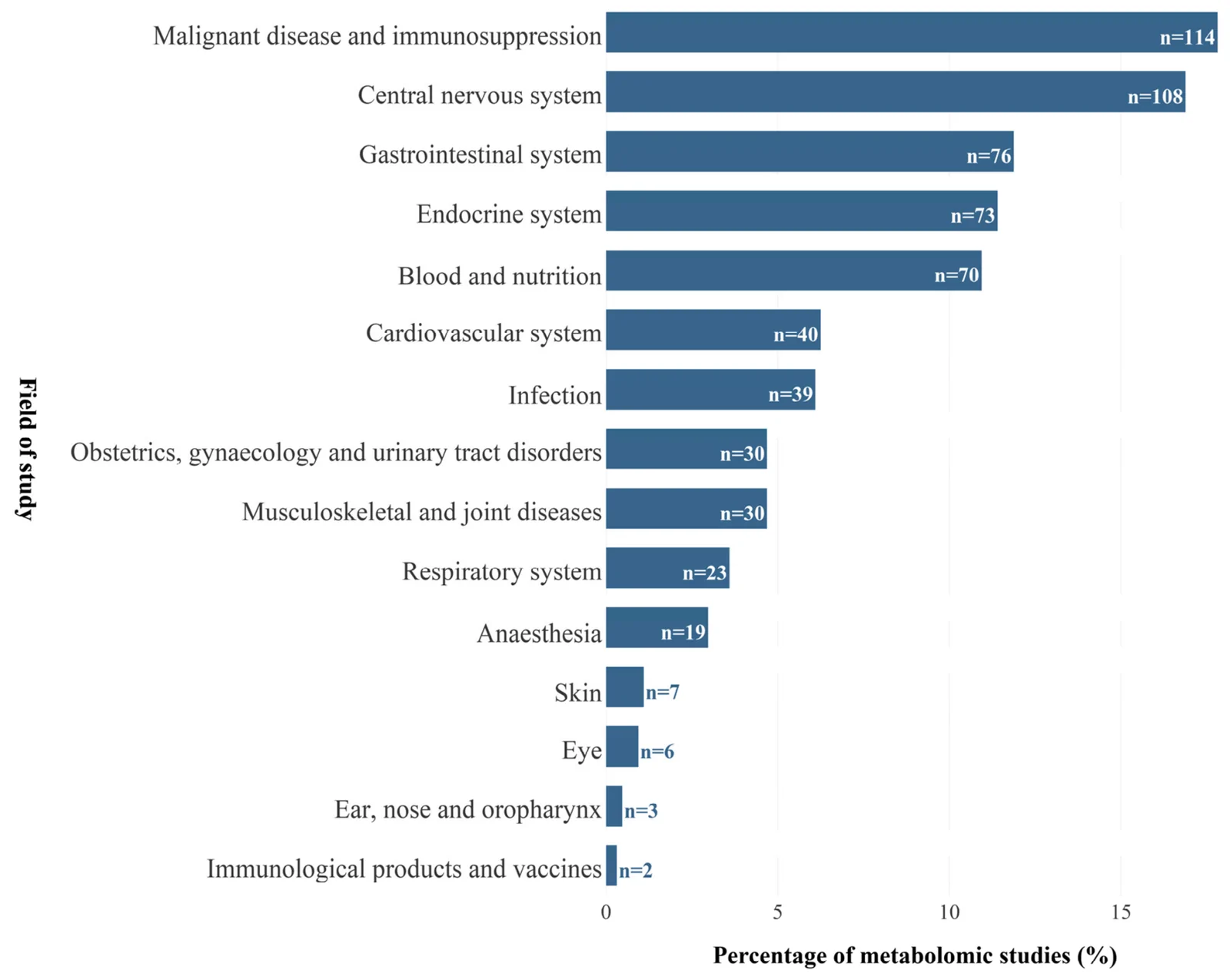
Distribution of metabolomic studies by therapeutic category.

The most common fields of metabolomic research (Figure 5) were malignant disease and immunosuppression (*n* = 114, 17.8%), followed by the central nervous system (*n* = 108, 16.9%), gastrointestinal system (*n* = 76, 11.9%), and endocrine system (*n* = 73, 11.4%).

Detailed datasets for all early-phase clinical trials and those incorporating metabolomics, including breakdowns by country, registry, year, phase, and outcome, are provided in the Supplementary Materials (Supplementary Tables S5–S9).

### 3.7. Manual Audit of Study Classifications and Methodological Characteristics

All 120 audited records described metabolomics or metabolite profiling and therefore met the broad inclusion criteria used in the primary analysis. However, 27 records (22.5%) described mass-balance or ADME studies in which profiling was restricted to the investigational drug and its metabolites. These records would not meet a more restrictive definition of metabolomics requiring the analysis of endogenous or broader metabolic profiles. Three records (2.5%) represented duplicate registrations of studies listed in more than one registry under slightly different titles, leaving 117 unique studies within the audited sample.

The biological specimen was specified in 93 records (77.5%). Blood was the most frequently reported specimen, appearing in 72 records (60.0%), followed by faeces in 36 (30.0%), urine in 25 (20.8%), cerebrospinal fluid in four (3.3%), and other specimens in four (3.3%). The other specimens comprised nasal turbinate secretions, skin swabs, tumour biopsy tissue and exhaled breath condensate. No specimen was explicitly reported in 27 records (22.5%). As individual records could include more than one specimen type, these categories were not mutually exclusive.

Reporting of the analytical platform and metabolomics approach was limited. Seven records (5.8%) specified LC–MS, one (0.8%) specified both LC–MS and NMR, and 112 (93.3%) did not specify the analytical platform. Five records (4.2%) described a targeted approach and seven (5.8%) an untargeted approach, while the remaining 108 (90.0%) did not specify the approach used. Consequently, the relative use of different analytical platforms and targeted versus untargeted metabolomics could not be reliably assessed.

## 4. Discussion

### 4.1. Principal Findings

Over the past 16 years, the incorporation of metabolomics in early-phase clinical trials has increased gradually but remains limited, accounting for 0.35% of all studies. Its use is concentrated predominantly within economically developed countries and within select therapeutic areas; particularly oncology, neurology, gastrointestinal disease and endocrinology. While China and the United States contribute the greatest number of metabolomic studies in absolute terms, reflecting their high trial volumes, the United Kingdom demonstrates the highest proportional integration. Metabolomics is more frequently used as a secondary, rather than a primary, outcome.

### 4.2. Modest Uptake of Metabolomics in Early-Phase Clinical Trials

While the published literature frequently highlights the expanding role of metabolomics in biomedical research, uptake within clinical trial design appears comparatively modest, suggesting ongoing barriers to implementation that need to be understood and addressed [3,29].

Cost is also likely to be an important barrier to wider adoption. Metabolomic analyses typically require expensive, highly specialised analytical platforms, such as mass spectrometry and nuclear magnetic resonance spectroscopy, together with substantial investment in infrastructure, maintenance and technical expertise [29]. Sample collection, handling, storage, and transport also present considerable logistical challenges [30]. To preserve metabolite integrity, biological specimens generally require rapid processing and storage at −80 °C, while repeated freeze–thaw cycles and haemolysis can substantially alter metabolite profiles [31,32]. Furthermore, where possible, samples are preferably analysed on the same analytical platform to minimise inter-instrument variation, creating additional challenges for large multicentre and international clinical trials that require centralised analysis. Finally, the lack of standardisation in metabolomic data acquisition, processing and analysis limits reproducibility and comparability between studies [33]. The high dimensionality of metabolomic datasets also necessitates advanced bioinformatic and statistical approaches, while the limited availability of appropriately trained specialists remains an additional barrier to implementation [16]. Moreover, many detected spectral features cannot be confidently identified, limiting biological interpretation and cross-study validation [34,35]. From a regulatory perspective, the validation of metabolomic biomarkers presents additional hurdles, with stringent requirements for standardisation and reproducibility before clinical acceptance [36,37]. Collectively, these factors limit the scalability of metabolomics and may explain its continued use predominantly as a secondary endpoint rather than a primary outcome in early-phase clinical trials.

### 4.3. Temporal Trends

Across the study period, metabolomics uptake in early-phase clinical trials increased in both absolute and proportional terms, extending the gradual upward trend previously reported by Burt and Nandal [17]. This overall growth likely reflects advances in high-throughput analytical platforms, computational methods and metabolite databases, together with increasing standardisation of metabolomic workflows [8].

The observed plateau in trial numbers following 2021 is consistent with previously reported global trends in clinical research activity which demonstrate a flattening in trial growth in the post–COVID-19 period [8,38,39]. This broader slowdown may partly explain the recent stabilisation in the number of metabolomic studies and is consistent with the plateau in PubMed-indexed metabolomics-related publications since 2021 reported by Stolaki et al. (2025) [8].

Most contributing registries were established prior to the study period, although a few including the Clinical Research Information Service of the Republic of Korea (February 2010), the Brazilian Clinical Trials Registry (16 December 2010), the EU Clinical Trials Register (23 March 2011), the Lebanese Clinical Trials Registry (25 July 2019), and the International Traditional Medicine Clinical Trial Registry (2019), were established during the study period [40,41,42]. Consequently, the observed increase in registered trials may partly reflect expansion of the ICTRP network and strengthening requirements for prospective trial registration, rather than an increase in metabolomics research alone.

### 4.4. Geographical Distribution

Although registry coverage and reporting practices may have influenced the observed geographical distribution, the concentration of metabolomics studies broadly aligned with established patterns of investment in multi-omic research infrastructure.

The United Kingdom’s leading position likely reflects sustained investment in integrated omics platforms and bioinformatics resources. This is exemplified by resources such as the UK Biobank’s Nightingale Health project; the largest metabolomic cohort globally, and MetaboLights, an open-access database hosted by the European bioinformatic institute in Cambridge [10,43,44]. China’s prominent contribution likely reflects both sustained government and industry investment and a strong research focus on omics [45]. Consequently, China is projected to experience substantial growth in metabolomics research over the coming decade [29,46].

This study benefits from the use of the WHO ICTRP, the most comprehensive source of international clinical trial information available. However, over 15 major national registries are omitted as they do not meet the WHO ICTRP inclusion criteria (Supplementary Table S2) [47]. Trials registered exclusively in these databases may therefore have been missed, potentially introducing geographical selection bias. The effect of these omissions is partly mitigated by overlap with included regional registries. For example, trials conducted in France and Spain may also be captured by the EU Clinical Trials Register or Clinical Trials Information System, while African trials may be registered with the Pan African Clinical Trials Registry. Nevertheless, the Russian, Saudi Arabian, Malaysian, Philippine, and Singaporean national registries may contain additional studies that are not reflected in ICTRP data. The extent of this under-ascertainment could not be quantified. The apparent geographical distribution may also be influenced by differences in national registration requirements and sponsors’ choice of registry.

### 4.5. Metabolomics Uptake by Therapeutic Area

Malignant disease and immunosuppression, central nervous system disorders, gastrointestinal disorders, and endocrine disorders were the most frequently represented therapeutic fields in our data. Despite methodological and classification differences, Burt and Nandal (2016) similarly identified endocrinology, oncology, cardiovascular disease, central nervous system disorders, immunology and gastroenterology among the principal therapeutic areas represented in metabolomics-related clinical trials [17].

Several biological and clinical factors may explain this distribution. In oncology, metabolic reprogramming is a hallmark of malignancy, creating opportunities for metabolomics to identify diagnostic and prognostic biomarkers and to monitor treatment response or toxicity [48]. In central nervous system disorders, metabolomics may support biomarker discovery and the stratification of clinically heterogeneous conditions, particularly where direct sampling of brain tissue is impractical. The gastrointestinal tract is also closely linked to the metabolome through interactions among diet, the intestinal microbiota and the host [49]. Faecal metabolomics provides a non-invasive measure of the metabolic products arising from these interactions; correspondingly, faeces were the second most frequently reported specimen after blood in our manual audit. Finally, the prominence of endocrine disorders may reflect the direct regulation of glucose, lipid, amino-acid, and energy metabolism by endocrine pathways, making metabolomics particularly relevant to conditions such as diabetes and obesity [50]. These biological and clinical applications may therefore contribute to the predominance of these therapeutic areas, although the distribution of trials may also reflect their broader representation in early-phase research.

### 4.6. Strengths and Weaknesses

This is the first study to use the WHO ICTRP dataset to evaluate metabolomics uptake in early-phase clinical trials. The scale and international coverage of the ICTRP, which aggregates data from 20 registries, enabled trends to be assessed over time and across geographical locations, trial phases, therapeutic fields and outcome types. A further strength was the manual review of record-level data, which assessed the robustness of study classification and quantified residual duplication and potential misclassification.

Nevertheless, the analysis remains subject to limitations inherent in aggregated registry data. Although WHO registry standards promote unambiguous trial identification, records of the same trial submitted to multiple registries are retained as separate entries in the full ICTRP dataset [35]. We therefore applied the deduplication procedure described above; however, because this relied on matching study titles, duplicates may have remained where the same trial was registered under different titles. Manual review identified residual duplication in 3 of 120 audited records (2.5%), suggesting that its overall effect was likely to be small. Registry data may also be missing or inconsistently reported, partly because a fully standardised vocabulary is not used across all fields. In this analysis, 5.5% of trials had missing or incomplete country information, consistent with previously reported rates of approximately 6% [47].

The metabolomics-related search terms were intentionally broad to maximise sensitivity but may have reduced specificity. In particular, the keyword “metabolite profil*” captured mass-balance and ADME studies in which mass spectrometry is typically used to characterise a parent drug and its metabolites. Although these studies involve metabolite profiling, they would not meet a more restrictive definition of metabolomics because they examine the disposition and biotransformation of a specific xenobiotic rather than endogenous or broader metabolic profiles. Such studies accounted for 22.5% of the audited sample, suggesting that the primary analysis may overestimate metabolomics uptake under a more restrictive definition. Their inclusion may also partly explain the relatively high number of phase 0 and phase I studies identified.

Finally, the available registry data limited more detailed characterisation of how metabolomics was applied. Most audited records did not report the analytical platform or whether the approach was targeted or untargeted, precluding meaningful comparisons of analytical methods. Classification by a single field of study may also have simplified multidisciplinary trials or interventions spanning several therapeutic domains. The findings should therefore be interpreted as describing broad patterns in the reported adoption of metabolomics rather than the methodological quality or precise analytical application of metabolomics within individual trials.

### 4.7. Implications and Future Directions

As metabolomics becomes increasingly important to precision medicine, its uneven global adoption may compound existing inequities in translational research, analogous to the underrepresentation of diverse populations in genomic studies [39,40]. Addressing this imbalance will require investment in analytical infrastructure, technical expertise, and international collaboration in underrepresented regions. Greater transparency is also needed: trial registries should encourage consistent reporting of the biological specimen, analytical platform, targeted or untargeted approach and, where applicable, chromatographic method used. More standardised reporting would facilitate comparisons between studies and enable future assessments to evaluate not only whether metabolomics was included, but also how it was applied.

The global metabolomics market is projected to grow from USD 2.52 billion in 2026 to USD 6.26 billion by 2034, driven partly by increasing applications in translational research [46]. Future adoption is likely to be supported by automated bioinformatic pipelines and advances in artificial intelligence and machine learning, which may improve the scalability and interpretation of complex metabolomic data. Continued development of resources such as the Human Metabolome Database, MetaboLights, and Metabolomics Workbench should further strengthen metabolite annotation, data sharing, and methodological standardisation, supporting the subsequent validation and integration of biomarkers into clinical trial design [10,11,12]. In parallel, initiatives such as the US National Institutes of Health’s *Multi-Omics for Health and Disease* programme are promoting the integration of metabolomics with other omics approaches, with the potential to strengthen its biological interpretation and clinical utility within precision medicine [51,52,53].

## 5. Conclusions

This study provides the first description of global metabolomics use in early-phase clinical trials. Despite its potential, metabolomics remains underutilised in this capacity, with variable uptake across countries, although use is gradually increasing. Overcoming technical, methodological, and regulatory barriers will be critical to fully realise the role of metabolomics in biomarker-driven, mechanism-informed clinical trial design and precision medicine.

## Data Availability

The data analysed in this study are available from the World Health Organization International Clinical Trials Registry Platform, subject to acceptance of its data-access terms. The complete code used for data cleaning and analysis is publicly available in a GitHub repository: https://github.com/Jack-Tandy-Thompson/Metabolomics-in-Early-Phase-Clinical-trials-from-2010-to-2025 (Version 1.0.0) accessed on 17 August 2026.

## Supplementary Materials

The following supporting information can be downloaded at: https://www.mdpi.com/article/10.3390/metabo16100777/s1, Table S1: ICTRP Data Providers; Table S2: National clinical trial registries not included in ICTRP; Table S3: Metabolomic search terms; Table S4: ICTRP data fields; Table S5: Stratified analysis by country; Table S6: Stratified analysis by phase; Table S7: Stratified analysis by registry; Table S8: Stratified analysis by year; Table S9: Stratified analysis by therapeutic indication of intervention; Table S10: Country look-up table.

## Abbreviations

The following abbreviations are used in this manuscript:

ICTRP	International Clinical Trials Registry Platform
WHO	World Health Organisation
UK	United Kingdom
US	United States of America
ADME	Absorption, Distribution, Metabolism, and Excretion
