# Supplementary material for: Metabolomics in Early-Phase Clinical Trials from 2010 to 2025
Source: Metabolites. 2026 Oct 14;16(10):777. doi: 10.3390/metabo16100777 (PMC13707337; doi:10.3390/metabo16100777)
Supplement: Supplementary file 1 [file metabolites-16-00777-s001.zip › metabolites-4540824-supplementary.pdf]

Supplementary Information to

Metabolomics in Early Phase Clinical Trials from 2010 to 2025

Table of contents **Page number**

Table S1. ICTRP Data Providers.......2

Table S2. National clinical trial registries not included in ICTRP.......3

Table S3. Metabolomic search terms.......4

Table S4. ICTRP datafields.......4

Table S5. Stratified analysis by country.......6

Table S6. Stratified analysis by phase.......14

Table S7. Stratified analysis by registry.......15

Table S8. Stratified analysis by year.......16

Table S9. Stratified analysis by therapeutic indication of intervention.......17

Table S10. Country look-up table.......18

Table S1. ICTRP Data providers

The 20 registries contributing to the ICTRP are shown together with their registry code prefixes and the dates on which they joined the platform. These dates indicate when each registry began contributing data to the ICTRP, rather than when the registry was established. The ICTRP may therefore contain studies with enrolment dates preceding a registry's date of accession. The EU Clinical Trials Register (EUCTR) and Clinical Trials Information System (CTIS) represent successive European Union registry systems and were combined for the purposes of this analysis.

Registry Name	Trial Registry code	Date Added to ICTRP
Australian New Zealand Clinical Trials Registry	ANZCTR	4th May 2007
Brazilian Clinical Trials Registry	REBEC	28th March 2011
Chinese Clinical Trial Register	ChiCTR	9th December 2008
Clinical Trials.gov	NCT	4th May 2007
Clinical Trials Registry-India	CTRI	9th December 2008
Clinical research Information Centre-Republic of Korea	CRiS	29th June 2010
Cuban Public Registry of clinical trials	RPCEC	5th April 2011
EU Clinical Trials Register	EUCTR	26th March 2012
Clinical Trials Information System	CTIS	1st May 2023
German Clinical Trials Register	DRKS	9th December 2008
Iranian Registry of Clinical Trials	IRCT	7th April 2009
ISRCTN.org	ISRCTN	4th May 2007
Japan Primary Registries Network	JPRN	16th February 2010
Pan African Clinical Trial Register	PACTR	25th September 2009
Peruvian Clinical Trials Register	REPEC	22nd July 2016
Sri Lanka Clinical Trials Register	SLCTR	10th March 2009
Thai Clinical Trials Register	TCTR	7th August 2013
The Netherlands National Trial Register	NL-OMON	9th December 2008
Lebanon Clinical Trials Registry	LBCTR	25th July 2019
International Traditional Medicine Clinical Trial Registry	ITMCTR	20th February 2023

Table S2. Registries worldwide that are **not** included in the ICTRP search portal.

These registries fail to meet specified WHO-ICTRP registry Network standards across 6 categories (‘content’, ‘quality & validity’, ‘accessibility’, ‘unambiguous identification’, ‘technical capacity’ and ‘Administration & governance’).

1. Argentina- Registro Nacional de Investigaciones en Salud
2. France- Essaid Cliniques-Santé.fr
3. Indonesia- Clinical Research Registry
4. Jordan- National Clinical Trials Registry
5. Malaysia-National Medical Research Register
6. Nepal- Clinical Trials Registry
7. Nigeria- Clinical Trials Registry
8. Pakistan- Clinical Trial Registry
9. Philippine Health Research Registry
10. Russian State Register of Medicines
11. Saudi Clinical Trials Registry
12. Singapore- Clinical Trials Registry
13. South African National Clinical Trials Register (SANCTR)
14. Spanish Register of Clinical Trials (REec)
15. Tanzania- Clinical Trials Register

Table S3. Metabolomic search terms. *Key search terms used to identify early phase clinical trials incorporating metabolomics within the ICTRP dataset, with their frequency of occurrence.*

Matched terms	n
1 "metabolom\\w*"	392
2 "metabolite profil\\w*"	148
3 "metabonom\\w*"	43
4 "lipidom\\w*"	20
5 "metabolom\\w*; metabonom\\w*"	13
6 "glycom\\w*"	10
7 "metabolom\\w*; lipidom\\w*"	8
8 "metabolom\\w*; metabolite profil\\w*"	4
9 "metabolom\\w*; pharmacometabolom\\w*"	2
**Total**	640

Table S4. ICTRP datafields. *Location of metabolomic search terms within the ICTRP dataset.*

Variables where key terms were found	n
1 secondary_outcomes	294
2 primary_outcome	123
3 public_title; scientific_title	70
4 scientific_title	37
5 primary_outcome; secondary_outcomes	29
6 public_title; scientific_title; primary_outcome	25
7 scientific_title; primary_outcome	13
8 public_title; scientific_title; secondary_outcomes	9
9 public_title	7

	10 scientific_title; secondary_outcomes	7
	11 public_title; scientific_title; primary_outcome; secondary_outcomes	4
	12 secondary_outcomes; conditions	4
	13 conditions	3
	14 interventions	2
	15 public_title; primary_outcome; secondary_outcomes	2
	16 public_title; scientific_title; secondary_outcomes; conditions	2
	17 public_title; conditions	1
	18 public_title; scientific_title; conditions	1
	19 public_title; scientific_title; interventions	1
	20 public_title; scientific_title; primary_outcome; conditions	1
	21 scientific_title; conditions	1
	22 scientific_title; interventions	1
	23 scientific_title; primary_outcome; secondary_outcomes	1
	24 scientific_title; secondary_outcomes; conditions	1
	25 secondary_outcomes; interventions	1
**Total**		640

Table S5. Metabolomic studies by country. *Weighted and absolute counts of metabolomic and all early phase clinical trials. Absolute counts assigned a value of one to each country listed per trial, whereas weighted counts apportioned a fraction of one to each country based on the total number of participating countries for each trial.*

	Continent	Country	Metabolomic		All Studies		Fraction met (weighted totals)	Percent met (weighted totals)
			Weighted total	Absolute counts	Weighted total	Absolute counts		
1	**South America**	Ecuador	1.00	1	6.23	19	0.1603966	16.03965977
2	**Europe**	Albania	0.56	3	4.45	22	0.1256634	12.56634314
3	**Africa**	Senegal	2.25	3	20.03	32	0.1123454	11.23454222
4	**Africa**	Burkina Faso	2.00	2	31.93	55	0.0626419	6.26419443
5	**Africa**	Rwanda	1.00	1	17.37	34	0.0575702	5.75701693
6	**Africa**	Gabon	1.00	1	29.11	46	0.0343543	3.43542954
7	**Asia**	Bangladesh	2.25	3	141.79	154	0.0158682	1.58681715
8	**South America**	Peru	1.10	2	84.21	525	0.013063	1.30629523
9	**Europe**	United Kingdom	76.38	88	5853.31	14033	0.0130497	1.30497345
10	**Africa**	Zambia	0.25	1	20.83	40	0.0120042	1.20042329
11	**Europe**	Norway	5.11	6	499.29	1577	0.0102368	1.02368034
12	**Africa**	South Africa	4.04	5	426.43	1453	0.0094704	0.94703698
13	**Europe**	Denmark	14.92	20	1620.43	4033	0.0092078	0.92078433
14	**Asia**	Indonesia	2.00	2	252.81	260	0.0079112	0.79112125
15	**Europe**	Holland	34.16	44	4682.40	9328	0.0072953	0.72953197
16	**Europe**	Finland	2.48	5	360.86	1395	0.0068619	0.68618534
17	**Asia**	China	236.04	237	39659.31	41675	0.0059517	0.59516527
18	**Europe**	Serbia	0.56	5	95.60	858	0.0058192	0.58191693
19	**Europe**	Lithuania	0.42	4	77.49	587	0.0054489	0.54488963
20	**Europe**	Hungary	2.35	12	495.94	3707	0.004748	0.47479707

**21**	**Europe**	Poland	4.84	14	1039.10	6416	0.0046549	0.46548796
**22**	**Asia**	Malaysia	1.00	1	223.09	729	0.0044826	0.44825641
**23**	**North America**	Canada	16.04	20	3604.11	10066	0.00445	0.44500447
**24**	**Europe**	Ukraine	0.84	7	188.53	1418	0.004439	0.44390267
**25**	**Asia**	Thailand	14.04	15	3321.28	4062	0.0042268	0.42268232
**26**	**Oceania**	Australia	17.81	21	4531.77	9783	0.003929	0.39289546
**27**	**Europe**	France	15.48	23	4356.03	12433	0.003554	0.35539976
**28**	**Europe**	Sweden	3.59	7	1124.22	3137	0.0031909	0.31909363
**29**	**Asia**	Pakistan	1.25	2	461.42	475	0.002709	0.27090239
**30**	**Europe**	Switzerland	2.28	4	866.68	2615	0.0026282	0.2628154
**31**	**Europe**	Greece	0.99	7	392.64	1941	0.0025317	0.25316885
**32**	**Europe**	Italy	7.42	17	3114.22	10801	0.0023813	0.2381287
**33**	**Asia**	South Korea	10.00	10	4217.90	7905	0.0023708	0.23708483
**34**	**Europe**	Germany	12.86	26	5570.84	14413	0.0023085	0.23085011
**35**	**Europe**	Estonia	0.14	1	62.86	502	0.0022727	0.22727033
**36**	**Europe**	Belgium	4.90	8	2253.43	7481	0.0021743	0.21742768
**37**	**Asia**	Taiwan	2.00	2	1016.14	3350	0.0019682	0.19682267
**38**	**North America**	United States	80.21	90	40922.67	53965	0.0019601	0.19600552
**39**	**Asia**	Singapore	1.00	1	510.54	1562	0.0019587	0.19587172
**40**	**Europe**	Spain	6.20	16	3374.76	13085	0.0018374	0.18373937
**41**	**Europe**	Ireland	0.36	2	207.14	983	0.0017433	0.17432912
**42**	**Europe**	Austria	1.54	3	899.64	3161	0.0017101	0.17100859
**43**	**Oceania**	New Zealand	1.33	2	843.62	2079	0.0015805	0.15804955
**44**	**South America**	Brazil	2.00	2	1385.47	3148	0.0014436	0.14435561
**45**	**Europe**	Romania	0.31	3	218.02	1584	0.0013995	0.13995377
**46**	**Asia**	Turkey	0.41	4	299.86	1835	0.0013788	0.13788321
**47**	**Europe**	Czech Republic	0.82	4	607.81	3819	0.0013528	0.13527668

**48**	**Asia**	Japan	8.04	9	6077.97	9274	0.0013226	0.13225575
**49**	**Europe**	Bulgaria	0.36	4	319.25	2095	0.0011298	0.11297783
**50**	**South America**	Chile	0.10	1	149.57	1034	0.0006686	0.06685932
**51**	**Europe**	Russia	0.29	3	671.56	2792	0.0004354	0.04354497
**52**	**South America**	Colombia	0.04	1	101.02	678	0.0003807	0.03807392
**53**	**Asia**	Israel	0.35	3	936.41	3312	0.0003738	0.03737683
**54**	**Asia**	India	5.04	6	13814.06	14644	0.0003647	0.03647343
**55**	**Europe**	Portugal	0.08	1	263.43	1374	0.0003163	0.03163342
**56**	**North America**	Mexico	0.14	2	464.44	1825	0.0002981	0.02981252
**57**	**Europe**	Slovakia	0.04	1	141.93	1070	0.000271	0.02709974
**58**	**Asia**	Iran	1.00	1	5748.78	5755	0.000174	0.01739501
**59**	**Africa**	Egypt	0.00	0	1569.60	1687	0	0
**60**	**South America**	Argentina	0.00	0	211.52	1922	0	0
**61**	**Africa**	Nigeria	0.00	0	184.88	199	0	0
**62**	**North America**	Cuba	0.00	0	165.63	169	0	0
**63**	**Africa**	Kenya	0.00	0	159.42	269	0	0
**64**	**Asia**	Saudi Arabia	0.00	0	125.55	231	0	0
**65**	**Asia**	Jordan	0.00	0	115.83	189	0	0
**66**	**Asia**	Vietnam	0.00	0	115.56	227	0	0
**67**	**Africa**	Uganda	0.00	0	113.12	194	0	0
**68**	**Asia**	Georgia	0.00	0	95.08	624	0	0
**69**	**Europe**	Moldova	0.00	0	92.94	434	0	0
**70**	**Asia**	Iraq	0.00	0	92.16	95	0	0
**71**	**Africa**	Tanzania	0.00	0	81.73	116	0	0
**72**	**Asia**	Lebanon	0.00	0	76.38	262	0	0
**73**	**Asia**	Philippines	0.00	0	73.85	372	0	0
**74**	**Europe**	Slovenia	0.00	0	70.09	282	0	0

**75**	**Africa**	Tunisia	0.00	0	68.35	126	0	0
**76**	**Asia**	Sri Lanka	0.00	0	62.09	87	0	0
**77**	**Europe**	Croatia	0.00	0	61.62	441	0	0
**78**	**Africa**	Ghana	0.00	0	59.24	85	0	0
**79**	**Europe**	Latvia	0.00	0	58.91	517	0	0
**80**	**Africa**	Ethiopia	0.00	0	54.63	57	0	0
**81**	**Africa**	Mali	0.00	0	52.48	65	0	0
**82**	**Europe**	Belarus	0.00	0	48.07	164	0	0
**83**	**North America**	Panama	0.00	0	39.01	133	0	0
**84**	**Asia**	United Arab Emirates	0.00	0	34.52	72	0	0
**85**	**North America**	Dominican Republic	0.00	0	31.86	57	0	0
**86**	**Africa**	Malawi	0.00	0	31.50	63	0	0
**87**	**Asia**	Cyprus	0.00	0	22.11	44	0	0
**88**	**Africa**	Congo	0.00	0	21.52	33	0	0
**89**	**Africa**	Gambia	0.00	0	20.58	29	0	0
**90**	**Asia**	Kazakhstan	0.00	0	20.17	33	0	0
**91**	**Africa**	Zimbabwe	0.00	0	20.13	63	0	0
**92**	**North America**	Antigua And Barbuda	0.00	0	19.42	31	0	0
**93**	**Europe**	Bosnia And Herzegovina	0.00	0	18.65	131	0	0
**94**	**Africa**	Sudan	0.00	0	17.67	22	0	0
**95**	**Asia**	Syria	0.00	0	17.17	21	0	0
**96**	**Asia**	Armenia	0.00	0	16.98	42	0	0
**97**	**North America**	Honduras	0.00	0	16.75	39	0	0
**98**	**Africa**	Cameroon	0.00	0	16.33	20	0	0
**99**	**Africa**	Côte D'Ivoire	0.00	0	15.41	31	0	0
**100**	**Asia**	Mongolia	0.00	0	15.08	17	0	0
**101**	**Asia**	North Korea	0.00	0	13.75	142	0	0

**102**	**Europe**	Luxembourg	0.00	0	13.54	45	0	0
**103**	**Europe**	Iceland	0.00	0	13.46	57	0	0
**104**	**North America**	Jamaica	0.00	0	13.07	24	0	0
**105**	**Asia**	Nepal	0.00	0	12.78	15	0	0
**106**	**North America**	Guatemala	0.00	0	11.78	105	0	0
**107**	**Asia**	Laos	0.00	0	11.45	14	0	0
**108**	**Africa**	Mauritius	0.00	0	11.26	49	0	0
**109**	**Africa**	Botswana	0.00	0	10.22	33	0	0
**110**	**North America**	El Salvador	0.00	0	10.21	23	0	0
**111**	**Asia**	Palestine	0.00	0	10.16	13	0	0
**112**	**Africa**	Guinea	0.00	0	10.01	14	0	0
**113**	**Asia**	Qatar	0.00	0	9.99	23	0	0
**114**	**Europe**	Macedonia	0.00	0	9.97	84	0	0
**115**	**Africa**	Mozambique	0.00	0	9.94	30	0	0
**116**	**South America**	Paraguay	0.00	0	9.92	26	0	0
**117**	**South America**	Bolivia	0.00	0	9.38	11	0	0
**118**	**Asia**	Myanmar	0.00	0	9.01	11	0	0
**119**	**Asia**	Kuwait	0.00	0	9.00	17	0	0
**120**	**Asia**	Oman	0.00	0	8.32	36	0	0
**121**	**Oceania**	Papua New Guinea	0.00	0	8.01	9	0	0
**122**	**Africa**	Sierra Leone	0.00	0	7.50	10	0	0
**123**	**Asia**	Cambodia	0.00	0	7.13	10	0	0
**124**	**North America**	Haiti	0.00	0	6.86	26	0	0
**125**	**Asia**	Uzbekistan	0.00	0	6.76	9	0	0
**126**	**South America**	Uruguay	0.00	0	6.51	8	0	0
**127**	**Africa**	Benin	0.00	0	6.35	11	0	0
**128**	**Africa**	Liberia	0.00	0	5.75	12	0	0

**129**	**North America**	Trinidad And Tobago	0.00	0	5.51	7	0	0
**130**	**North America**	Nicaragua	0.00	0	5.34	7	0	0
**131**	**South America**	Venezuela	0.00	0	5.26	22	0	0
**132**	**North America**	Bahamas	0.00	0	5.21	9	0	0
**133**	**North America**	Costa Rica	0.00	0	4.99	43	0	0
**134**	**Africa**	Morocco	0.00	0	4.74	26	0	0
**135**	**Asia**	Azerbaijan	0.00	0	4.44	7	0	0
**136**	**Asia**	Kyrgyzstan	0.00	0	4.03	5	0	0
**137**	**Africa**	Madagascar	0.00	0	3.50	5	0	0
**138**	**Asia**	Afghanistan	0.00	0	3.21	5	0	0
**139**	**Asia**	Yemen	0.00	0	3.17	7	0	0
**140**	**Africa**	Equatorial Guinea	0.00	0	3.01	4	0	0
**141**	**Asia**	Bahrain	0.00	0	2.63	10	0	0
**142**	**Africa**	Algeria	0.00	0	2.23	15	0	0
**143**	**Africa**	Burundi	0.00	0	2.01	3	0	0
**144**	**Asia**	Timor-Leste	0.00	0	2.01	3	0	0
**145**	**Europe**	Monaco	0.00	0	2.00	3	0	0
**146**	**Africa**	Angola	0.00	0	1.82	6	0	0
**147**	**Europe**	Malta	0.00	0	1.54	8	0	0
**148**	**North America**	Barbados	0.00	0	1.51	5	0	0
**149**	**Africa**	Somalia	0.00	0	1.17	5	0	0
**150**	**Asia**	Bhutan	0.00	0	1.01	2	0	0
**151**	**Africa**	Togo	0.00	0	1.01	2	0	0
**152**	**Africa**	Comoros	0.00	0	1.00	1	0	0
**153**	**Africa**	Eritrea	0.00	0	1.00	1	0	0
**154**	**Oceania**	Federated States Of Micronesia	0.00	0	1.00	1	0	0
**155**	**Europe**	Kosovo	0.00	0	1.00	1	0	0

**156**	**Africa**	South Sudan	0.00	0	1.00	1	0	0
**157**	**North America**	Belize	0.00	0	0.50	1	0	0
**158**	**Oceania**	Kiribati	0.00	0	0.50	1	0	0
**159**	**Oceania**	Palau	0.00	0	0.34	2	0	0
**160**	**Europe**	Liechtenstein	0.00	0	0.31	2	0	0
**161**	**Africa**	Eswatini	0.00	0	0.26	6	0	0
**162**	**Africa**	Libya	0.00	0	0.17	4	0	0
**163**	**Europe**	Montenegro	0.00	0	0.16	3	0	0
**164**	**Africa**	Mauritania	0.00	0	0.16	3	0	0
**165**	**Asia**	Brunei	0.00	0	0.06	1	0	0
**166**	**Africa**	Central African Republic	0.00	0	0.01	1	0	0
**167**	**Africa**	Chad	0.00	0	0.01	1	0	0
**168**	**South America**	Guyana	0.00	0	0.01	1	0	0
**169**	**Oceania**	Marshall Islands	0.00	0	0.01	1	0	0
**170**	**Africa**	Namibia	0.00	0	0.01	1	0	0
**171**	**Oceania**	Nauru	0.00	0	0.01	1	0	0
**172**	**Africa**	Niger	0.00	0	0.01	1	0	0
**173**	**North America**	Saint Kitts And Nevis	0.00	0	0.01	1	0	0
**174**	**North America**	Saint Lucia	0.00	0	0.01	1	0	0
**175**	**North America**	Saint Vincent And The Grenadin es	0.00	0	0.01	1	0	0
**176**	**Oceania**	Samoa	0.00	0	0.01	1	0	0
**177**	**Europe**	San Marino	0.00	0	0.01	1	0	0
**178**	**Oceania**	Solomon Islands	0.00	0	0.01	1	0	0
**179**	**South America**	Suriname	0.00	0	0.01	1	0	0
**180**	**Asia**	Tajikistan	0.00	0	0.01	1	0	0
**181**	**Oceania**	Tonga	0.00	0	0.01	1	0	0

**182**	**Asia**	Turkmenistan	0.00	0	0.01	1	0	0
**183**	**Oceania**	Tuvalu	0.00	0	0.01	1	0	0
**184**	**Oceania**	Vanuatu	0.00	0	0.01	1	0	0
		Missing data	25.00	25	9804.42	10081	0.0025499	0.25498697
		**Total**	640.00	814	183041	327333		

Table S6. Breakdown by phase of trial

Clinical phase	Metabolomic studies (n)	Percent of all metabolomic studies (%)	All studies (n)	Percent metabolomic (%)
**Phase 2**	183	28.6	86061	0.2126
**Phase 1/2**	41	6.4	20371	0.2013
**Phase 1**	229	35.8	56633	0.4044
**Phase 0**	187	29.2	19976	0.9361

Table S7. Stratified analysis by registry

	Registry	Metabolomic studies	All Studies	Percent metabolomic (%)
1	Clinical Trials.gov (USA)	235	96196	0.244293
2	Chinese Clinical Trial Register	186	24324	0.764677
3	EU Clinical Trials Register/Clinical Trials Information System	82	18692	0.43869
4	Clinical Trials Registry India	4	13547	0.029527
5	Japan Primary Registries Network	20	8944	0.223614
6	Iranian Registry of Clinical Trials	1	5539	0.018054
7	Thai Clinical Trials Register	13	3574	0.363738
8	Australian New Zealand Clinical Trials Registry	18	3103	0.580084
9	The Netherlands National Trial Register	17	2075	0.819277
10	International Traditional Medicine Clinical Trial Registry	32	1761	1.817149
11	ISRCTN (UK)	17	1328	1.28012
12	Clinical research information centre (Republic of Korea)	4	1263	0.316706
13	Pan African Clinical Trial Register	5	937	0.533618
14	German Clinical Trials Register	4	780	0.512821
15	Brazilian Clinical Trials Registry	1	542	0.184502
16	Cuban Public Registry of Clinical Trials	0	163	0
17	Peruvian Clinical Trials Register	1	149	0.671141
18	Sri Lanka Clinical Trials Register	0	72	0
19	Lebanese Clinical Trials Registry	0	52	0
	**Total**	640	183041	

Table S8. Stratified analysis by year

Year	Metabolomic studies	All studies	Metabolomic percentage
2010	9	7145	0.125962
2011	10	7281	0.137344
2012	11	7301	0.150664
2013	14	7441	0.188147
2014	15	8075	0.185759
2015	16	8491	0.188435
2016	29	9072	0.319665
2017	30	9382	0.319761
2018	31	10312	0.300621
2019	52	12663	0.410645
2020	54	15828	0.341168
2021	82	16931	0.484319
2022	71	15548	0.45665
2023	81	15670	0.516911
2024	84	16619	0.505446
2025	51	15282	0.333726
**Total**	640	183041	0.34964844

Table S9. Stratified analysis of metabolomic studies by therapeutic indication of intervention

Field of study	Metabolomic studies (n)	Percentage (%)
Malignant disease and immunosuppression	114	17.8
Central nervous system	108	16.9
Gastrointestinal system	76	11.9
Endocrine system	73	11.4
Blood and nutrition	70	10.9
Cardiovascular system	40	6.25
Infection	39	6.09
Musculoskeletal and joint diseases	30	4.69
Obstetrics, gynaecology and urinary tract disorders	30	4.69
Respiratory system	23	3.59
Anaesthesia	19	2.97
Skin	7	1.09
Eye	6	0.938
Ear, nose and oropharynx	3	0.469
Immunological products and vaccines	2	0.312

Table S10. Country Look-up table for cleaning inconsistently entered country names into structured inputs

**Inputted Host Country Name**	**Standardised country name (United Nations Member states)**
**Czechia**	Czech Republic
**Czech**	Czech Republic
**Russian Federation**	Russia
**Russian Fed.**	Russia
**Iran, Islamic Republic of**	Iran
**Iran (Islamic Republic Of)**	Iran
**Hong Kong**	China
**United States of America**	United States
**Us**	United States
**United States Minor Outlying Islands**	United States
**The United States of America**	United States
**The United States**	United States
**The Us**	United States
**United State**	United States
**Unitedstates Of America**	United States
**Perto Rico**	United States
**United Status**	United States
**U.s,A**	United States

**United States,**	United States
**Unitedstates Ofamerica**	United States
**United States Of Americ**	United States
**Usa (Including Puerto Rico)**	United States
**Puerto Rico**	United States
**Guam**	United States
**United Staes**	United States
**Usa**	United States
**U.s.a**	United States
**U.s.**	United States
**U.s.a.**	United States
**American Samoa**	United States
**Holland, The Netherlands**	Holland
**Netherland**	Holland
**Nederland**	Holland
**Republic Of Netherlands**	Holland
**Netherlands**	Holland
**Netherlands Kingdom Of The**	Holland
**Nertherlands**	Holland
**Thenetherlands**	Holland
**The Netherlands**	Holland

**Netherlands Antilles**	Holland
**Aruba**	Holland
**Moldova, Republic of**	Moldova
**Republic Of Moldova**	Moldova
**Moldova Republic Of**	Moldova
**Modalvia**	Moldova
**Western Sahara**	Morocco
**Turkey (Türkiye)**	Turkey
**Turkey (Turkiye)**	Turkey
**Türkiye**	Turkey
**United Republic of Tanzania**	Tanzania
**Tanzania, United Republic Of**	Tanzania
**Korea, Democratic People’s Republic of**	North Korea
**Democratic People's Republic Of Korea**	North Korea
**Korea, Democratic People's Republic Of**	North Korea
**Korea North**	North Korea
**Democratic People Republic Of Korea**	North Korea
**Democratic People's Republic Of**	North Korea
**Syrian Arab Republic**	Syria
**Phillipines**	Philippines
**None**	Missing data

**??**	Missing data
**À¹„À¸—À¸¢**	Missing data
**Not Applicable**	Missing data
**No**	Missing data
**Other**	Missing data
**-**	Missing data
**Non**	Missing data
**Not Stated**	Missing data
**The People's Republic Of**	Missing data
**Many**	Missing data
**Noe**	Missing data
**None.**	Missing data
**Nore**	Missing data
**Nothing**	Missing data
**Refer To The Section, Other.**	Missing data
**A**	Missing data
**N**	Missing data
**North America, Europe, Asia (Except Japan), Oceania, South America, Africa**	Missing data
**Apac**	Missing data
**Part A: Usa Part B C: Asia, North America, Eu**	Missing data

**East Asia(Taiwan, Hong Kong, Singapore And Other)**	Missing data
**Asia Exept Japan**	Missing data
**Asia**	Missing data
**Emea**	Missing data
**Expansion Phase :**	Missing data
**Null**	Missing data
**Asia/ Europe/ North America/ Oceania/ European Union / South America**	Missing data
**Japan,Asia(Except Japan)**	Missing data
**Japan,Asia(Except Japan),North America**	Missing data
**Dose Escalation Phase :**	Missing data
**Japan,Asia(Except Japan),Europe**	Missing data
**Japan,Europe**	Missing data
**Japan,North America**	Missing data
**Japan,North America,South America**	Missing data
**Japan,South America**	Missing data
**America**	Missing data
**European Union**	Missing data
**Africa**	Missing data
**Asia(Except Japan),Africa**	Missing data
**South America**	Missing data

**North America**	Missing data
**Europe**	Missing data
**Oceania**	Missing data
**Nate**	Missing data
**Asia(Except Japan)**	Missing data
**Asia Except Japan**	Missing data
**Japan, Asia(Exept Japan)**	Missing data
**Benelux**	Missing data
**Congo, The Democratic Republic Of The**	Congo
**Democratic Republic Of The Congo**	Congo
**Congo - Kinshasa**	Congo
**Republic Of The Congo**	Congo
**Lao People's Democratic Republic**	Laos
**Chnia**	China
**P.r. China**	China
**P.r.china**	China
**Beijing**	China
**Cnina**	China
**Chain**	China
**Chine**	China
**Guangdong**	China

**Hina**	China
**People’s Republic Of China**	China
**People's Republic Of China**	China
**Shanghai**	China
**Republic of China**	China
**Anhui**	China
**Chaina**	China
**Chengdu**	China
**Chiina**	China
**Chin**	China
**Chin’a**	China
**China Hong Kong**	China
**China Mainland (Prc)**	China
**China·**	China
**Ching**	China
**Chinia**	China
**Chins**	China
**Chna?Cna**	China
**Chia**	China
**Cina**	China
**Chna**	China

**Can**	China
**Guangxi Zhuang Autonomous Region**	China
**Hebei**	China
**Hksar**	China
**Hong Kong Sar, China**	China
**Hong Kong, China**	China
**Hong Kong,China**	China
**Maternal and Child Health Hospital of Changxing Couty**	China
**Pepole's Republic Of China**	China
**Rpc**	China
**Tianjin**	China
**Zhong Guo**	China
**Zhongguo**	China
**Hongkong**	China
**Macau**	China
**Chian**	China
**Chinese**	China
**The First Affiliated Hospital Of Hunan University Of Traditional Chinese Medicine**	China
**The People's Republic Of China**	China
**Pr China**	China

**Cna**	China
**Cote Divoire**	Côte D'Ivoire
**Cote D’Ivoire**	Côte D'Ivoire
**Côte D'ivoire**	Côte D'Ivoire
**Macedonia, The Former Yugoslav Republic of**	Macedonia
**North Macedonia**	Macedonia
**The Former Yugoslav Rep Of Macedonia**	Macedonia
**The Former Yugoslav Republic Of Macedonia**	Macedonia
**Viet Nam**	Vietnam
**Korea, South**	South Korea
**Korea South**	South Korea
**Korea Republic Of**	South Korea
**Korea (The Republic Of)**	South Korea
**Sounth Korea**	South Korea
**Korea (Republic Of)**	South Korea
**Sourth Korea**	South Korea
**Korea,Republic Of**	South Korea
**Suoth Korea**	South Korea
**S Korea**	South Korea
**Sk**	South Korea
**Sout Korea**	South Korea

**Southkorea**	South Korea
**S. Korea**	South Korea
**Rep. Of Korea**	South Korea
**Korea**	South Korea
**Koria**	South Korea
**Korea, Republic Of**	South Korea
**Republic Of Korea,**	South Korea
**Republic Of Korea**	South Korea
**Sount Korea**	South Korea
**Rupublic Of Korea**	South Korea
**United Kindgdom**	United Kingdom
**Isle of Man**	United Kingdom
**United Kingdom Of Great Britain And Northern Ireland**	United Kingdom
**United Kingdom Of Great Britain**	United Kingdom
**The United Kingdom**	United Kingdom
**U.k.**	United Kingdom
**United Kingdum**	United Kingdom
**Unitedkingdom Of Great Britain**	United Kingdom
**United Kingdom Of Great Britain And Northern Irel**	United Kingdom
**United Of Kingdom**	United Kingdom

**Unitred Kingdom**	United Kingdom
**Scotland**	United Kingdom
**Wales**	United Kingdom
**Northern Ireland**	United Kingdom
**England**	United Kingdom
**Gibraltar**	United Kingdom
**Uk**	United Kingdom
**Cayman Islands**	United Kingdom
**Japan Only**	Japan
**None (Japan Only)**	Japan
**Palestinian Territory, Occupied**	Palestine
**Palestinian Territories**	Palestine
**Palestinian Territories, Occupied**	Palestine
**Thai**	Thailand
**Maetha**	Thailand
**Thailand.**	Thailand
**Thailan**	Thailand
**Thaiand**	Thailand
**Aland Islands**	Finland
**Republic Of Finland**	Finland
**Mone**	Myanmar

**México**	Mexico
**Republic Of Mexico**	Mexico
**Taiwan R.o.c**	Taiwan
**Taiwan Province Of China**	Taiwan
**Taiwan,Province Of China**	Taiwan
**Taiwian**	Taiwan
**Taiwan, Province Of China**	Taiwan
**Taiwan (Province Of China)**	Taiwan
**Venezuela, Bolivarian Republic Of**	Venezuela
**Venezuela (Bolivarian Republic Of)**	Venezuela
**Malaysa**	Malaysia
**Malasya**	Malaysia
**Serbia And Montenegro**	Serbia
**Former Serbia and Montenegro**	Serbia
**Republic Of Serbia**	Serbia
**Isreal**	Israel
**State of Irael**	Israel
**Israelc**	Israel
**State Of Israel**	Israel
**Italia**	Italy
**Republic Of Italy**	Italy

**Itary**	Italy
**Spein**	Spain
**Spain,**	Spain
**Kingdom Of Spain**	Spain
**Sweden Etc**	Sweden
**Sweeden**	Sweden
**Swizerland**	Switzerland
**The Gambia**	Gambia
**Canda**	Canada
**Columbia**	Colombia
**Paraguar**	Paraguay
**Hungaria**	Hungary
**Republic Of Portugal**	Portugal
**Portugal,**	Portugal
**Bulugaria**	Bulgaria
**Commonwealth Of Australia**	Australia
**Australia**	Australia
**Christmas Island**	Australia
**Australia,**	Australia
**Reunion**	France
**Rèunion**	France

**Réunion**	France
**Federal Republic Of Germany**	Germany
**German**	Germany
**Kingdom Of Belgium**	Belgium
**Beugium**	Belgium
**Berugium**	Belgium
**Republic Of Singapore**	Singapore
**Shingapore**	Singapore
**Latovia**	Latvia
**Ukrine**	Ukraine
**Ausria**	Austria
**Austraria**	Austria
**Republic Of South Africa**	South Africa
**Bolivia, Plurinational State Of**	Bolivia
**Libyan Arab Jamahiriya**	Libya
**Arabia Saudi**	Saudi Arabia
**Bosnial And Herzegovina**	Bosnia And Herzegovina
**Lebano**	Lebanon
**Roumania**	Romania
**Newzealand**	New Zealand
**Niue**	New Zealand

**Slovakia,**	Slovakia
**Brunei Darussalam**	Brunei
**Faroe Islands**	Denmark
**Greenland**	Denmark
**French Guiana**	France
**Martinique**	France
**Guadeloupe**	France
**Swaziland**	Eswatini
